# Generalized lymphatic anomalies and review of the current management landscape: a case report and review of the literature

**DOI:** 10.1186/s13256-021-02953-9

**Published:** 2021-08-10

**Authors:** Tao Liu, Sana Basseri, Ben Mussari, Dominique DaBreo, Sandip SenGupta, Dalila Villalobos, Sara Awad

**Affiliations:** 1 Department of Medicine, Queen’s University, C3-008, 166 Brock Street, Kingston, ON K7L 5G2 Canada; 2 Department of Radiology, Queen’s University, Kingston, ON Canada; 3 Department of Pathology, Queen’s University, Kingston, ON Canada

**Keywords:** Generalized lymphatic anomalies, Lymphatic malformations, Diffuse systemic lymphangiomatosis, Multisystemic lymphangiomatosis, Lymphangioma, Generalized vascular anomalies, Case report

## Abstract

**Background:**

Generalized lymphatic anomaly previously known as diffuse systemic lymphangiomatosis is a rare multisystem congenital disease arising from the lymphatic system, and it is characterized by abnormal proliferation of the lymphatic channels in osseous and extraosseous tissues. It typically affects children or young adults. Although it is benign, it can be misdiagnosed as malignancy because of its diffuse and debilitating nature depending on the site of involvement. Due to its rarity, diagnosis is often delayed, leading to potential significant morbidity or mortality if vital organs are involved. Furthermore, its potential for multiorgan involvement with no curative treatment makes its management challenging.

**Case presentation:**

We describe a case of a 35-year-old Caucasian female, who presented with epigastric pain and was subsequently extensively investigated at multiple tertiary centers by numerous specialists for query malignancy and metabolic bone disorder following incidental computed tomography imaging findings of multiple osteolytic lesions in the axial skeleton, and low-attenuating lesions in the axilla, spleen, and mediastinum. The diagnosis was confirmed with an axillary excisional biopsy. She was clinically stable with no end organ damage. She was monitored conservatively.

**Conclusions:**

The case illustrates the importance of increased awareness among clinicians for this rare congenital disease to enable earlier diagnosis and to avoid unnecessary invasive investigations. Furthermore, this case highlights the potential need for multiple biopsies of affected sites to confirm diagnosis. We also discuss the emergence of interferon therapy, chemotherapy, immunosuppression, and immunotherapy as medical management for this condition.

## Background

Generalized lymphatic anomaly (GLA) is a rare nonneoplastic congenital condition characterized by abnormal proliferation of lymphatic vessels resulting in dilated and abnormally connected thin-walled lymphatic channels [1]. It can be localized involving a single organ, or more commonly generalized affecting multiple organs with the most common being the lungs and bones [2]. It typically presents in children and young adults in the first two decades of life [2, 3]. Its incidence is unknown given its rarity, which poses diagnostic and therapeutic challenges.

Treatment for GLA is mostly supportive with no curative intent. Multiple treatment modalities have been trialed in the literature, but there are no randomized trials to date to determine standard of treatment given its rarity. Complicating matters is the broad and inconsistent nomenclature in the literature, which includes GLA and diffuse systemic lymphangiomatosis. However, emerging data in treatment options are promising.

In this case report, we describe a previously healthy 35-year-old Caucasian female with incidental findings of multiple axial skeletal osteolytic lesions, and multisystemic hypoattenuating lesions on computed tomography (CT). The diagnosis was confirmed histologically by axillary excisional biopsy. She was managed conservatively with close monitoring.

## Case presentation

A 35-year-old Caucasian female was referred to endocrinology for assessment and management of query malignancy and metabolic bone disease. She was otherwise healthy and had recently relocated to our center. She was taking pantoprazole for suspected gastroesophageal reflux disease but had otherwise no known chronic medical conditions. Her only other medications were oral contraceptive pill, calcium, and vitamin D3.

On detailed history, she initially presented to her family physician 9 months prior with a 1-year history of nonspecific epigastric pain. She denied any constitutional symptoms including fever, chills, or night sweats. However, she reported a remote episode of 10-lb weight loss that she attributed to personal stress, which she regained by the time she was assessed in the endocrinology clinic. She denied bone or muscle pain, previous fragility fractures or vertebral fractures, and changes in bowel or urinary habits and stated that her menstrual cycles were regular. She smoked marijuana regularly for the last 15 years and denied alcohol intake or tobacco use. Her physical examination was significant for a palpable lymph node in her left axilla, which was soft and immobile. The rest of her physical examination was largely unremarkable, with normal cardiovascular, respiratory, abdominal, and screening endocrinological exam for thyroid nodules and cushingoid appearance. She had no family history of metabolic bone disorder. Her epigastric pain was felt secondary to gastroesophageal reflux disease, and she was started on a trial of pantoprazole.

Her initial blood work with her family physician revealed hemoglobin of 137 g/L, leukocytes of 5.6 × 10^9^/L with normal differential, platelets of 192 × 10^9^/L, creatinine of 55 μmol/L, sodium of 143 mmol/L, potassium of 3.9 mmol/L, chloride of 107 mmol/L, sodium bicarbonate of 26 mmol/L, aspartate aminotransferase (AST) of 12 U/L, alanine aminotransferase (ALT) of 14 U/L, alkaline phosphatase (ALP) of 94 U/L, lactate dehydrogenase (LDH) of 123 U/L, and mild elevation of both pancreatic enzymes amylase and lipase. The elevated pancreatic enzymes prompted a CT scan of her abdomen, which showed no radiologic evidence of pancreatitis, but revealed multiple other findings including innumerable low-density splenic lesions, a horseshoe kidney, and multiple osteolytic lesions throughout the axial skeleton. These findings prompted referrals to hematology, medical oncology, and endocrinology for query malignancy and metabolic bone disease.

Further blood work from consultants revealed absence of monoclonal antibodies on serum and urine electrophoresis, thyroid stimulating hormone (TSH) of 0.83 mIU/L, parathyroid hormone (PTH) of 3.7 pmol/L, cancer antigen 15-3 (CA 15-3) of 10 kU/L, carcinoembryonic antigen (CEA) of 2.8 μg/L, and a normal Papanicolaou test. Blood work was remarkable for elevated free kappa light chains of 26.42 mg/L with upper limit of normal of 19.6 mg/L, elevated kappa-to-lambda ratio of 2.14 with upper limit of normal of 1.65, and low 25-hydroxyvitamin D3 level at 21 nmol/L (reference range 75–150 nmol/L).

**Fig. 1:**
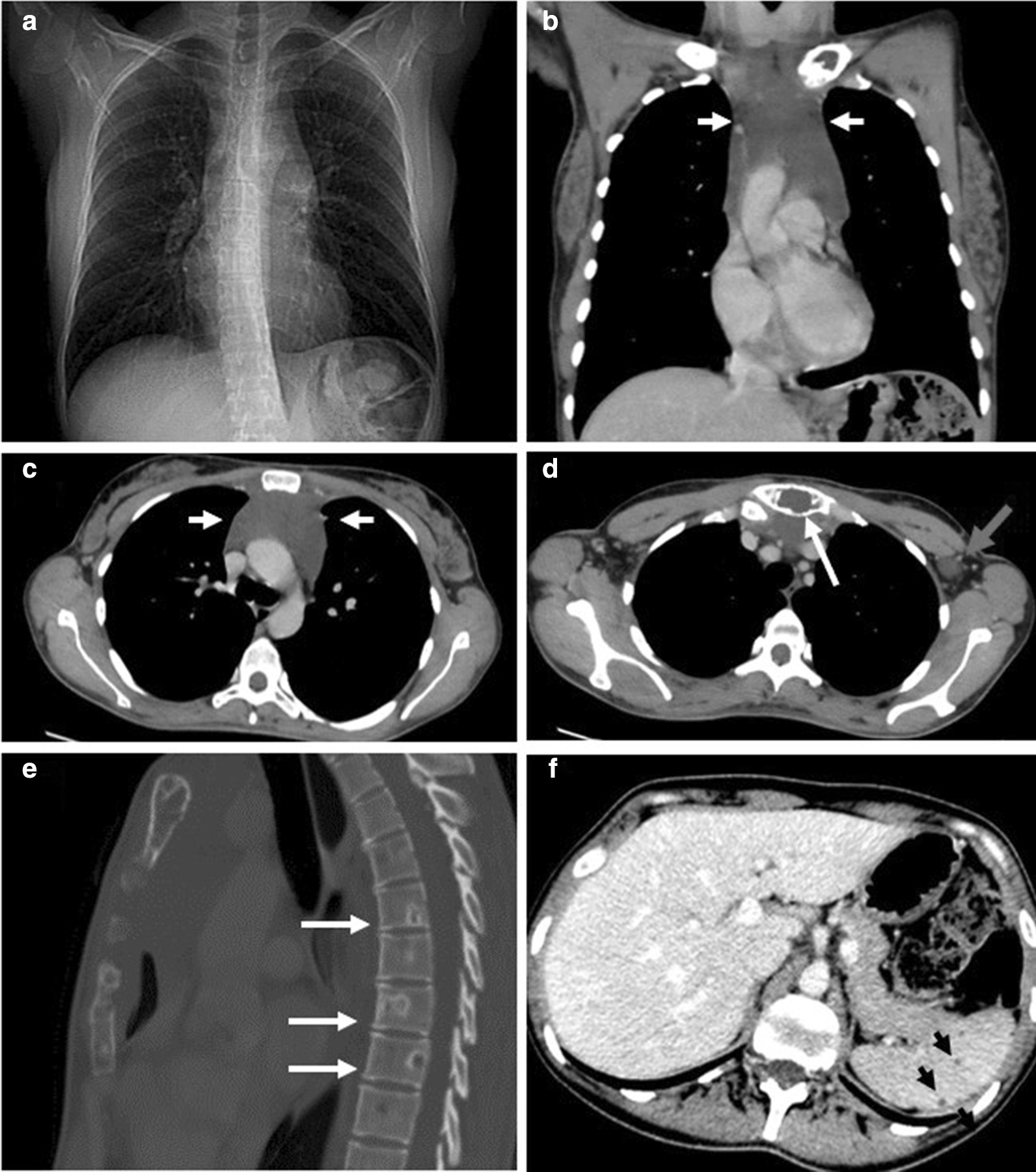
Contrast-enhanced CT chest demonstrating a mediastinal mass and osteolytic bone lesions. **A** Scout film from a CT chest showing widening of the mediastinum. **B**, **C** Contrast-enhanced CT chest coronal and axial views demonstrate a large anterior mediastinal low attenuating mass extending to the thoracic outlet (white arrows). **D** CT axial view through the superior mediastinum showing a left axillary low-attenuating lesion (grey arrow) and a well-defined osteolytic lesion in the manubrium (white arrow). **E** Sagittal view from the CT on bone window demonstrates numerous well-defined osteolytic lesions with sclerotic margins (white arrows) in multiple vertebral bodies. **F** Axial CT of the upper abdomen demonstrating numerous small splenic hypoattenuating lesions (black arrows)

**Fig. 2:**
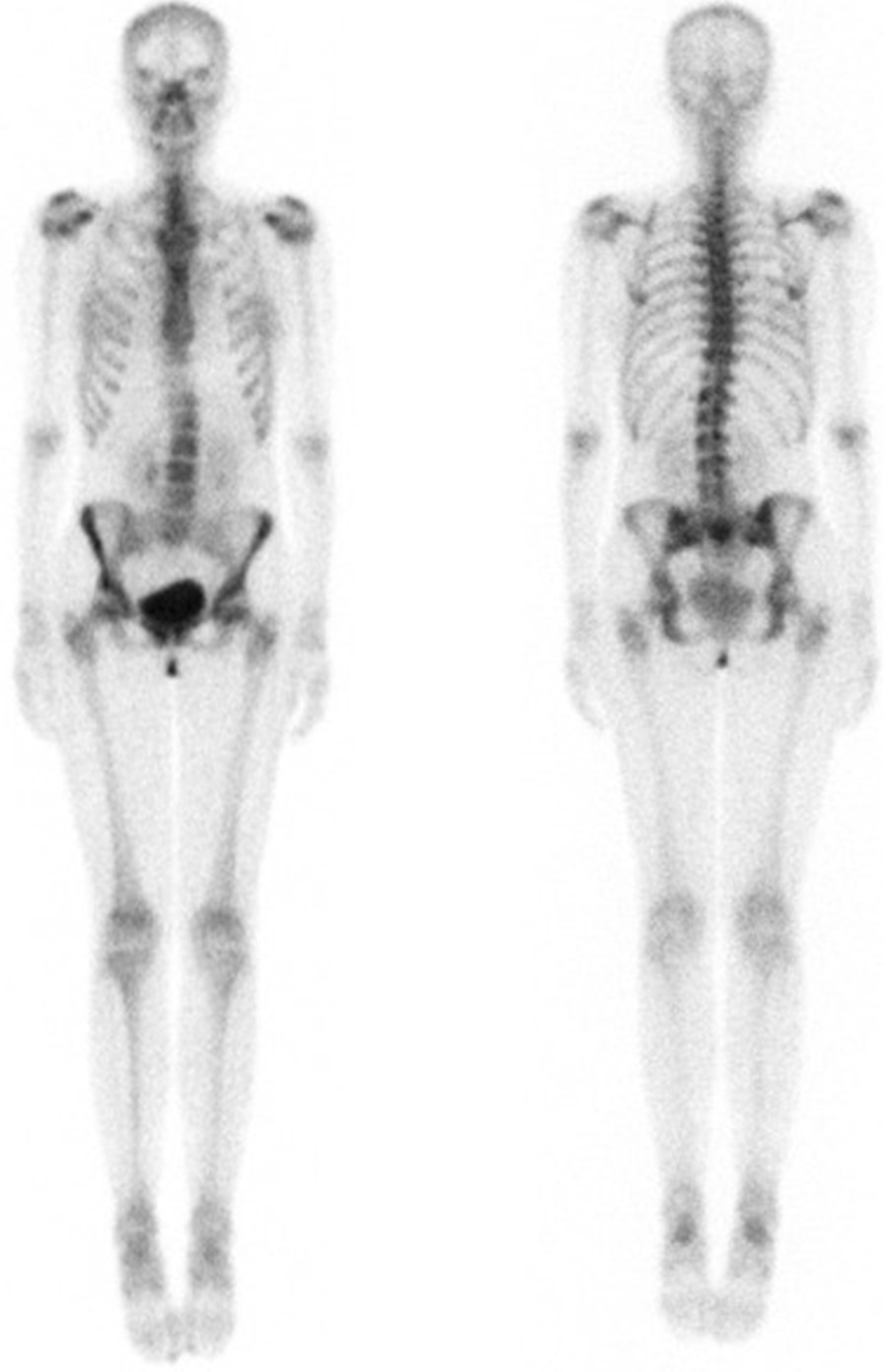
Whole body Tc99m-methylene diphosphonate (MDP) scan. There were no scintigraphic correlates to the numerous osteolytic lesions seen on CT and MRI. There was no evidence of hypermetabolic osseous metastases

**Fig. 3:**
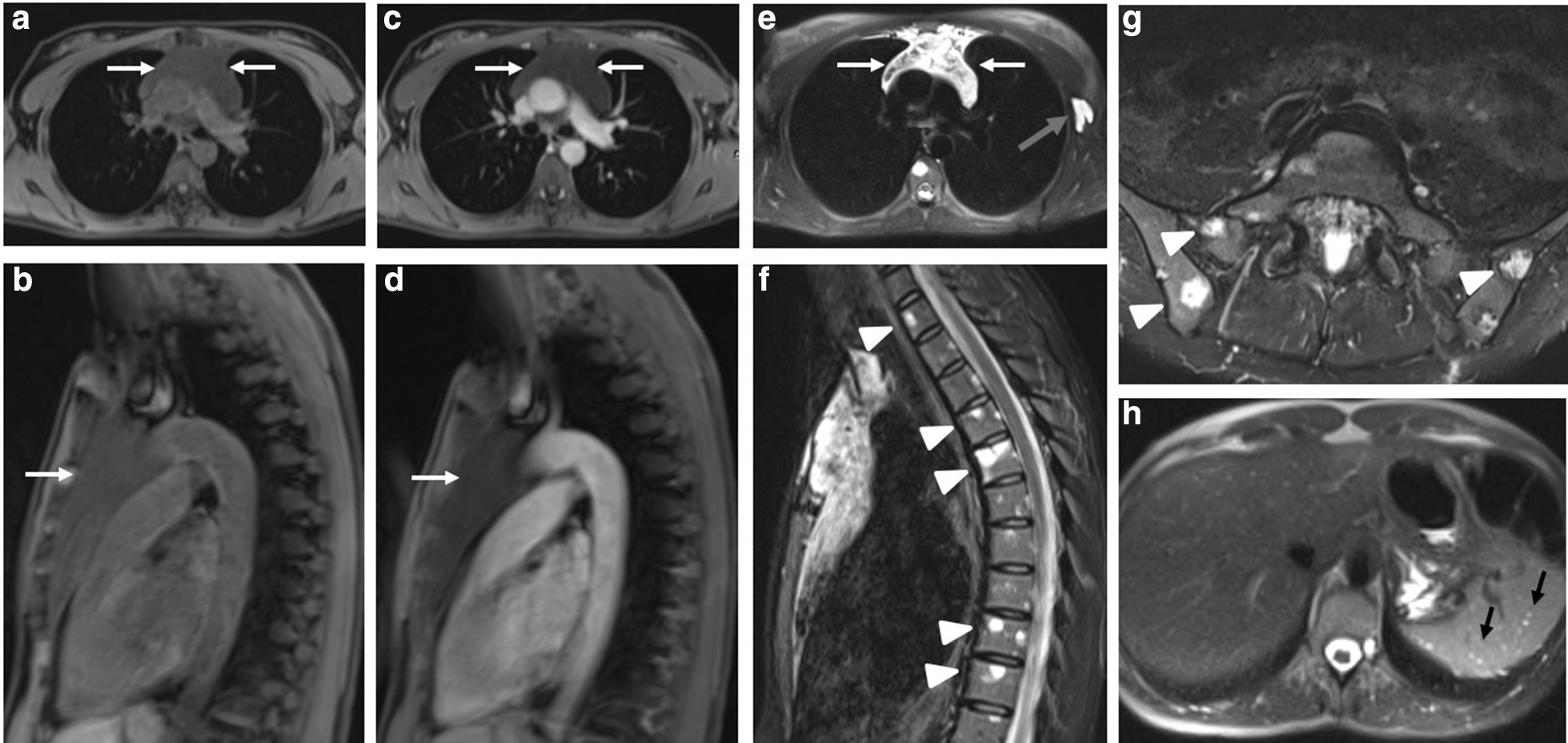
MRI demonstrating a large cystic mass in the anterior mediastinum (white arrows), a cystic lesion in the left axilla (grey arrow), as well as numerous bone and splenic lesions (white arrowheads). Axial and Sagittal T1 fat-saturated images prior to administration of gadolinium (**A**, **B**) and post-injection of gadolinium (**C**, **D**) demonstrate no features of enhancement of the mediastinal mass. Axial HASTE fat-saturated sequence (**E**), sagittal STIR (**F**), and axial T2 fat-saturated sequence (**G**) demonstrate cystic nature of mediastinal mass, axillary mass, and multiple hyperintense bone lesions involving the vertebra and iliac bones. **H** Axial HASTE fatsaturated sequence of the upper abdomen illustrates numerous small splenic hyperintense lesions (black arrows)

Further imaging including a contrast-enhanced chest CT demonstrated a 6.5 × 13.1 × 4.9 cm circumscribed homogeneous hypodense soft tissue mass occupying the anterior mediastinum extending from the thoracic inlet to just inferior to the thyroid, multiple tiny lung hypointensities in a peribronchovascular distribution with slight upper lobe predominance, a 1.8 cm low-attenuating lesion in the inferior margin of the left axilla, numerous hypointense splenic lesions, and, again, numerous osteolytic lesions in the axial skeleton (Fig. 1). Of these osteolytic lesions, some had sclerotic margins with cortical breach at the manubrium. This was further investigated with a bone scan that found no scintigraphic evidence of radiotracer uptake corresponding to the numerous bone lesions (Fig. 2). Skeletal survey was also done, which redemonstrated the lytic lesions in the axial skeleton, with the most prominent lesion noted in the pelvis. Magnetic resonance imaging (MRI) of the chest confirmed a cystic mass in the mediastinum, without evidence for enhancement following administration of gadolinium (Fig. 3A–D). MRI also confirmed a cystic lesion in the left axilla (Fig. 3E) and showed multifocal well-circumscribed T2 hyperintense bone lesions in the spine and pelvis (Fig. 3F, G). There was no evidence of spinal cord compression or fractures seen on MRI. Additionally, MRI demonstrated multiple T2 hyperintense splenic lesions, which were too small to characterize but were suggestive of cystic lymphangiomas (Fig. 3H). A bone mineral density study was obtained, and bone density and Z-score were within the expected range for her age- and gender-matched control. Thyroid ultrasound showed a sub-centimeter left thyroid nodule with no suspicious features of malignancy. She also received a mammogram that showed no evidence of suspicious breast lesions.

**Fig. 4:**
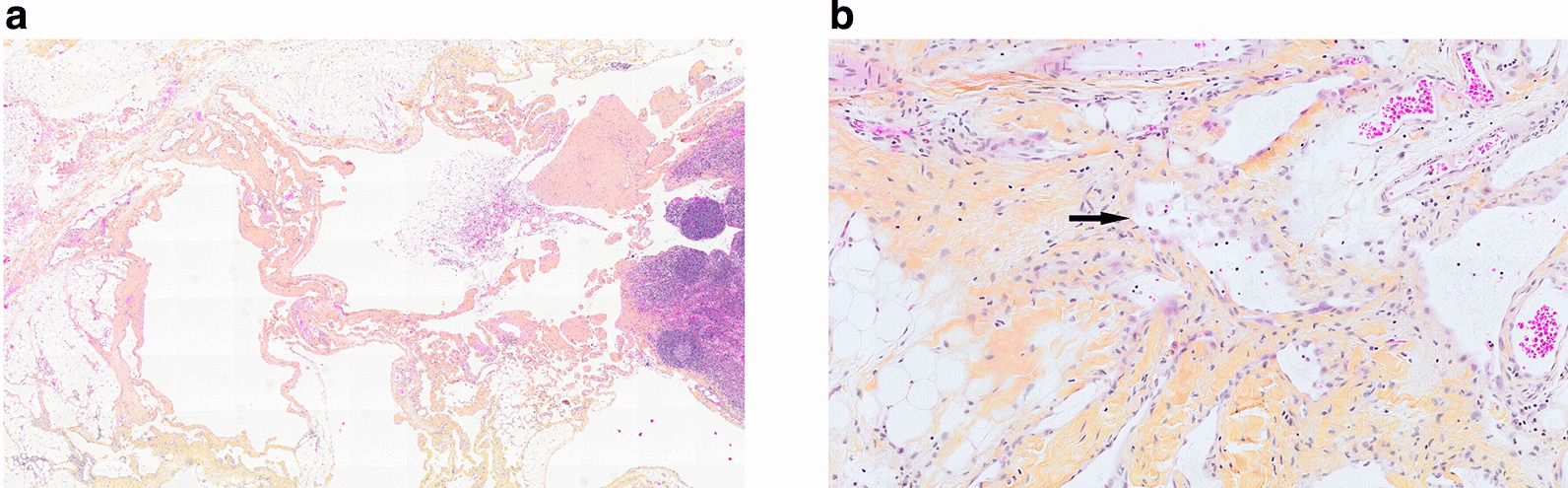
Histology of the left axillary cystic lesion. **A** Dilated and cystic structures in the perinodal adipose tissue (low-power magnification). **B** Attenuated endothelial cells lining with no cytologic atypia indicated by the black arrow (high-power magnification)

Full upper and lower endoscopies were performed with biopsies taken, all of which were negative for malignancy. She was evaluated by the thoracic surgery service at an outside institution for a CT guided fine-needle aspiration (FNA) biopsy of the mediastinal mass. The results ruled out malignancy, but the biopsy was not available for review. Subsequently, she was assessed by the orthopedics service, and an open bone biopsy of the left iliac crest was performed. This showed normal bone marrow cells with no primary or secondary malignancy. General surgery was consulted for an excisional biopsy of the left axillary lesion to confirm the diagnosis. Pathological examination showed characteristic features including dilated and cystic structures lined by endothelial cells with no atypia or evidence of malignancy (Fig. 4). Taken together, the constellation of radiological findings on CT and MRI in addition to the pathology findings confirmed the diagnosis of GLA.

She continued to do well on follow-up, and her epigastric pain had resolved on a course of pantoprazole. She did not suffer from any fractures or other local symptoms related to her GLA and had no respiratory symptoms despite the mediastinal mass. She was also counseled about the importance of bone health measures with adequate calcium in the diet and vitamin D3 supplements as well as regular exercise to strengthen her bones. She was counseled to seek medical attention if she developed symptoms, with a plan to follow up in clinic yearly.

## Discussion and conclusions

Since 2014, the term “diffuse systemic lymphangiomatosis” has been replaced with GLA to distinguish it from multiple other congenital lymphatic malformations [4]. This group of lymphatic anomalies includes Gorham–Stout disease (GSD), which is differentiated from GLA by its progressive osteolysis and bone cortex invasion [5, 6]. GLA is a rare condition arising from abnormal development of lymphatic vessels resulting in thin-walled dilated lymphatic channels that are abnormally interconnected and could result in symptoms related to obstruction and invasion of the adjacent vital structures. A previous case series of 53 patients with thoracic lymphangiomatosis demonstrated that 49% presented with pleural effusion, 45% with pulmonary infiltrates, and 39% with bone lesions [2]. It is debated whether there is an equal distribution between genders. Alvarez *et al*. described a 74% male predominance of thoracic lymphangiomatosis, while Schuster *et al*. claimed an equal distribution [2, 3]. Although it is benign, GLA can be debilitating because of invasion and/or compression of the surrounding structures [2]. The prognosis is poor in children, with a mortality rate of 39% in children less than 16 years of age, whereas mortality is 0% in adults [2].

The etiology of this condition is unclear, but it is thought to be caused by mutations affecting lymphoid proliferation during intrauterine development before the 20th week of gestation leading to formation of benign interconnected cysts containing lymph fluid [7]. Many patients present in childhood or early adulthood. Earlier disease is typically more aggressive and carries a profoundly higher mortality rate as patients die from multiorgan failure as a result of lymphatic invasion to vital organs [2].

Diagnosis is often delayed as it is usually discovered incidentally when patients present with unrelated symptoms such as those seen in our patient or when a complication occurs such as fragility fractures. The first clue is usually classical imaging features, suspected by an experienced radiologist. GLA affects all the skeletal and extraskeletal organs (except the brain) where there are lymphatic channels. It can involve the skeleton, lungs, mediastinum, spleen, liver, kidney, colon, and retroperitoneum [5, 8–12]. When it involves multiple organ systems, it can mimic advanced solid or hematologic malignancy or metastasis [5]. Plain radiographic imaging features of GLA includes radiolucent bone lesions, which may progress to bone “disappearance,” fragmentation, and fracture, which has been described in GSD [13]. A chest radiograph may also demonstrate chylous pleural effusions, mediastinal widening, or soft tissue masses [14]. Whole body CT scan is important to determine the extent of organ involvement. CT findings may reveal well-demarcated osteolytic bone lesions with a sclerotic margin, which can affect multiple bones in proximity [5, 15]. These features were also corroborated by our case, with involvement of multiple vertebra and iliac bones. Soft tissue and parenchymal organ involvement in GLA present as multicystic nonenhancing masses with microlobulated margins and internal septation [5, 9]. Additionally, mesenteric or pulmonary interstitial thickening, pulmonary nodules, and pleural/pericardial effusions may be present [14]. Ultimately, GLA can be diagnosed based on complete history, physical examination, and radiographical findings. However, when biopsy is necessary, a site that maximizes the likelihood of diagnosis and is most amendable is pursued, which may be extraskeletal. When a skeletal biopsy is done, it shows bony changes along with abnormal lymphatic channels that stain positively for PROX-1 and D2-40 [1, 4]. Interestingly, open bone biopsy of left iliac crest in our case did not demonstrate these findings. Rather, the excisional biopsy of the left axillary cystic lesion was diagnostic.

Treatment of this condition is conservative and targeted towards symptom management and complications if they develop. Currently, there are no curative treatments, and management is primarily focused on relieving symptoms and managing complications. Multiple treatment modalities have been proposed in the literature [16–31]. For asymptomatic solitary lesions, observation alone is reasonable. For symptomatic solitary lesions, treatment with surgery, radiotherapy, or laser therapy may be warranted [16–19]. However, for disseminated disease, the role of surgery is often limited to biopsy to establish diagnosis or symptomatic treatment for effusions (for example, excision, sclerotherapy, radiotherapy, pleurodesis) [16–19]. Alternatively, many modalities of medical treatment have been proposed. These include interferon therapy, chemotherapy, immunosuppression, and immunotherapy [20–31].

Retrospective and prospective studies have shown that mechanistic target of rapamycin (mTOR) inhibitors, namely sirolimus, can induce a partial response resulting in reduction of disease burden, leading to improvements in quality of life [6, 20–22]. mTOR is a serine/threonine kinase in the PI3K/AKT pathway that governs cellular growth, proliferation, and angiogenesis. By inhibiting mTOR, sirolimus can reduce bony disease and pericardial and pleural effusions, and improve other quality-of-life measures [6, 20–22]. Adams *et al*. conducted a phase 2 clinical trial enrolling 61 patients with confirmed vascular anomalies of whom 7 had GLA. Of the seven GLA patients, all had a 100% partial response defined as reduction in the size of the lesion by 20%, improvement in end organ dysfunction, or improvement in self-reported quality-of-life score [6].

Alternatively, multiple case reports have investigated tyrosine kinase inhibitors [23, 24]. Rossler *et al*. utilized a regimen of sunitinib and taxol for two patients, one with GLA presenting with respiratory failure and the other with lymphatic malformation in GSD presenting with back pain and swelling [23]. Sunitinib is a tyrosine kinase inhibitor in the ras/raf/MAPK pathway, which governs angiogenesis. Sunitinib was observed to result in both clinical and radiographical improvements [23]. Subsequently, Libby *et al*. used imatinib for a patient with progressive pulmonary disease who failed sirolimus and sorafenib, an inhibitor of vascular endothelial growth factor (VEGF) [24]. Imatinib was observed to resolve the patient’s dyspnea and hemoptysis [24]. Notably, inhibition of tyrosine kinase may be an alternative to or work in a synergistic fashion to sirolimus [23]. Further research is needed to delineate the role of each for treatment of GLA [23].

Pegylated interferon alpha has also been reported as a potential treatment modality in various case reports [25, 26]. The proposed mechanism is through inhibition of proliferation and angiogenesis by downregulation of VEGF [25]. Ozeke *et al*. reviewed 14 pediatric cases with progressive lymphangiomatosis, in which all but one presented with pulmonary involvement. All were treated with interferon alpha, resulting in clinical improvement in almost all patients [27]. Similarly, a more recent case series by Venkatramani *et al*. looked at eight cases of GSD and found that all patients treated with interferon alpha and bisphosphonates experienced stabilization of bone disease [28].

Another promising treatment modality was published by Grunewald *et al*. on their findings of bevacizumab—a monoclonal anti-VEGF antibody—for the treatment of lymphatic malformations [29]. Grunewald *et al*. documented complete cessation of osteolysis and stable disease even at follow-up after 27 months [28]. Furthermore, bevacizumab may also be used in the adult population [30, 31]. Two additional case reports have since been published that demonstrated objective clinical improvements in patients given bevacizumab for diffuse pulmonary lymphangiomatosis [30, 31]. Onyeforo *et al*. treated a 51-year-old man with diffuse pulmonary lymphangiomatosis and reported improvement in lung function including FEV1, FVC, and DLCO [30]. Meanwhile, Aman *et al*. treated a 40-year-old female, leading to resolution of hemoptysis and radiographical stability [31].

In summary, GLA is a rare congenital condition that can involve multiple organ systems except the brain. Diagnosis is often challenging and delayed owing to its nonspecific presentation and resemblance to malignancy. It is diagnosed with a complete clinical assessment and classical radiological features. When indicated, the diagnosis can be confirmed with skeletal or extraskeletal tissue biopsy. As we gain a deeper understanding of potential molecular targets for treatment, there is increased optimism to provide a better quality of life for these patients. Management is conservative and directed towards symptoms and/or complications as a result of the disease. Although there is no standard of treatment or cure, emerging evidence for interferon therapy, chemotherapy, immunosuppression, and immunotherapy are all promising. More research in therapeutics will be required to improve the knowledge base to optimally treat this unusual condition.

## Data Availability

Not applicable.

## Abbreviations

ALP: Alkaline phosphatase
ALT: Alanine aminotransferase
AST: Aspartate aminotransferase
CA 15-3: Cancer antigen 15-3
CEA: Carcinoembryonic antigen
CT: Computed tomography
DLCO: Diffusion capacity of carbon monoxide
FEV1: Forced expiratory volume in 1 s
FNA: Fine-needle aspiration
FVC: Forced vital capacity
GLA: General lymphatic anomalies
GSD: Gorham–Stout disease
LDH: Lactate dehydrogenase
MRI: Magnetic resonance imaging
PTH: Parathyroid hormone
TSH: Thyroid stimulating hormone
VEGF: Vascular endothelial growth factor

## References

[1] Adams DM, Ricci KW. Vascular anomalies: diagnosis of complicated anomalies and new medical treatment options. Hematol Oncol Clin. 2019;33(3):455–70. 10.1016/j.hoc.2019.01.011 31030813

[2] Alvarez OA, Kjellin I, Zuppan CW. Thoracic lymphangiomatosis in a child. J Pediatr Hematol Oncol. 2004;26(2):136–41. 10.1097/00043426-200402000-00018 14767208

[3] Schuster SR, Gang DL. Case 30–1980: an 11-year-old girl with multiple osteolytic lesions and a mediastinal mass. N Engl J Med. 1980;303(5):270–6.

[4] Wassef M, Blei F, Adams D, Alomari A, Baselga E, Berenstein A, Burrows P, Frieden IJ, Garzon MC, Lopez-Gutierrez JC, Lord DJ. Vascular anomalies classification: recommendations from the International Society for the Study of Vascular Anomalies. Pediatrics. 2015;136(1):e203–14. 10.1542/peds.2014-3673 26055853

[5] Kwag E, Shim SS, Kim Y, Chang JH, Kim KC. CT features of generalized lymphangiomatosis in adult patients. Clin Imaging. 2013;37(4):723–7. 10.1016/j.clinimag.2012.12.003 23391872

[6] Adams DM, Trenor CC, Hammill AM, Vinks AA, Patel MN, Chaudry G, Wentzel MS, Mobberley-Schuman PS, Campbell LM, Brookbank C, Gupta A. Efficacy and safety of sirolimus in the treatment of complicated vascular anomalies. Pediatrics. 2016;137(2):e20153257. 10.1542/peds.2015-3257 PMC4732362 26783326

[7] Faul JL, Berry GJ, Colby TV, Ruoss SJ, Walter MB, Rosen GD, Raffin TA. Thoracic lymphangiomas, lymphangiectasis, lymphangiomatosis, and lymphatic dysplasia syndrome. Am J Respir Crit Care Med. 2000;161(3):1037–46. 10.1164/ajrccm.161.3.9904056 10712360

[8] Lin RY, Zou H, Chen TZ, Wu W, Wang JH, Chen XL, Han QX. Abdominal lymphangiomatosis in a 38-year-old female: case report and literature review. World J Gastroenterol. 2014;20(25):8320. 10.3748/wjg.v20.i25.8320 PMC4081712 25009412

[9] Kumar P, Kumar S, Husain N, Chandra A. Isolated cystic lymphangiomatosis of spleen in an adult: a diagnostic conundrum. Case Rep. 2018;2018:bcr-2017. 10.1136/bcr-2017-223856 PMC5893968 29622712

[10] Satria MN, Pacheco-Rodriguez G, Moss J. Pulmonary lymphangiomatosis. Lymphat Res Biol. 2011;9(4):191–3. 10.1089/lrb.2011.0023 PMC3246407 22196284

[11] Jung SW, Cha JM, Lee JI, Joo KR, Choe JW, Shin HP, Kim KY. A case report with lymphangiomatosis of the colon. J Korean Med Sci. 2010;25(1):155–8. 10.3346/jkms.2010.25.1.155 PMC2800000 20052363

[12] Siderits R, Ouattara O, Abud A, Moubarak I, McIntosh N, Godyn J. Retroperitoneal cystic abdominal lymphangiomatosis diagnosed by fine needle aspiration: a case report. Acta Cytol. 2009;53(2):191–4. 10.1159/000325123 19365974

[13] Damron TA, Brodke DS, Heiner JP, Swan JS, DeSouky S. Case report 803: Gorham’s disease (Gorham–Stout syndrome) of scapula. Skeletal Radiol. 1993;22(6):464–7. 10.1007/BF00538454 8248825

[14] Marom EM, Moran CA, Munden RF. Generalized lymphangiomatosis. Am J Roentgenol. 2004;182(4):1068. 10.2214/ajr.182.4.1821068 15039189

[15] Kotecha R, Mascarenhas L, Jackson HA, Venkatramani R. Radiological features of Gorham’s disease. Clin Radiol. 2012;67(8):782–8. 10.1016/j.crad.2012.01.009 22424931

[16] Elluru RG, Balakrishnan K, Padua HM. Lymphatic malformations: diagnosis and management. In: Seminars in pediatric surgery 2014, Vol. 23, No. 4. WB Saunders. pp. 178–85. 10.1053/j.sempedsurg.2014.07.002 25241095

[17] Balakrishnan K, Menezes MD, Chen BS, Magit AE, Perkins JA. Primary surgery vs primary sclerotherapy for head and neck lymphatic malformations. JAMA Otolaryngol Head Neck Surg. 2014;140(1):41–5. 10.1001/jamaoto.2013.5849 24288004

[18] Morgan P, Keller R, Patel K. Evidence-based management of vascular malformations. Facial Plast Surg. 2016;32(02):162–76. 10.1055/s-0036-1581141 27097138

[19] Chen W, Adams D, Patel M, Gupta A, Dasgupta R. Generalized lymphatic malformation with chylothorax: long-term management of a highly morbid condition in a pediatric patient. J Pediatr Surg. 2013;48(3):e9-12. 10.1016/j.jpedsurg.2013.01.017 23480949

[20] Ricci KW, Hammill AM, Mobberley-Schuman P, Nelson SC, Blatt J, Bender JL, McCuaig CC, Synakiewicz A, Frieden IJ, Adams DM. Efficacy of systemic sirolimus in the treatment of generalized lymphatic anomaly and Gorham–Stout disease. Pediatr Blood Cancer. 2019;66(5):e27614. 10.1002/pbc.27614 PMC6428616 30672136

[21] Gurskytė V, Zeleckienė I, Maskoliūnaitė V, Mickys U, Šileikienė V. Successful treatment of diffuse pulmonary lymphangiomatosis with sirolimus. Respir Med Case Rep. 2020;29:101014. 10.1016/j.rmcr.2020.101014 PMC7013175 32071853

[22] Dvorakova V, Rea D, O’Regan GM, Irvine AD. Generalized lymphatic anomaly successfully treated with long-term, low-dose sirolimus. Pediatr Dermatol. 2018;35(4):533–4. 10.1111/pde.13494 29582448

[23] Rössler J, Saueressig U, Kayser G, von Winterfeld M, Klement GL. Personalized therapy for generalized lymphatic anomaly/Gorham–Stout disease with a combination of sunitinib and taxol. J Pediatr Hematol/Oncol. 2015;37(8):e481. 10.1097/MPH.0000000000000436 PMC4617281 26458155

[24] Libby LJ, Narula N, Fernandes H, Gruden JF, Wolf DJ, Libby DM. Imatinib treatment of lymphangiomatosis (generalized lymphatic anomaly). J Natl Compr Cancer Netw. 2016;14(4):383–6. 10.6004/jnccn.2016.0045 27059187

[25] Timke C, Krause MF, Oppermann HC, Leuschner I, Claviez A. Interferon alpha 2b treatment in an eleven-year-old boy with disseminated lymphangiomatosis. Pediatr Blood Cancer. 2007;48(1):108–11. 10.1002/pbc.20461 16007599

[26] Laverdière C, David M, Dubois J, Russo P, Hershon L, Lapierre JG. Improvement of disseminated lymphangiomatosis with recombinant interferon therapy. Pediatr Pulmonol. 2000;29(4):321–4. 10.1002/(sici)1099-0496(200004)29:4<321::aid-ppul13>3.0.co;2-c 10738021

[27] Ozeki M, Funato M, Kanda K, Ito M, Teramoto T, Kaneko H, Fukao T, Kondo N. Clinical improvement of diffuse lymphangiomatosis with pegylated interferon alfa-2b therapy: case report and review of the literature. Pediatr Hematol Oncol. 2007;24(7):513–24. 10.1080/08880010701533603 17786787

[28] Venkatramani R, Ma NS, Pitukcheewanont P, Malogolowkin MH, Mascarenhas L. Gorham’s disease and diffuse lymphangiomatosis in children and adolescents. Pediatr Blood Cancer. 2011;56(4):667–70. 10.1002/pbc.22948 21298758

[29] Grunewald TG, Damke L, Maschan M, Petrova U, Surianinova O, Esipenko A, Konovalov D, Behrends U, Schiessl J, Wörtler K, Burdach S. First report of effective and feasible treatment of multifocal lymphangiomatosis (Gorham–Stout) with bevacizumab in a child. Ann Oncol. 2010;21(8):1733–4. 10.1093/annonc/mdq331 20605931

[30] Onyeforo E, Barnett A, Zagami D, Deller D, Feather I. Diffuse pulmonary lymphangiomatosis treated with bevacizumab. Respirol Case Rep. 2019;7(1):e00384. 10.1002/rcr2.384 PMC6260917 30510764

[31] Aman J, Thunnissen E, Paul MA, van Nieuw Amerongen GP, Vonk-Noordegraaf A. Successful treatment of diffuse pulmonary lymphangiomatosis with bevacizumab. Ann Intern Med. 2012;156(11):839–40. 10.7326/0003-4819-156-11-201206050-00016 22665821

